# Colon penetration by a femoral prosthesis: a case report

**DOI:** 10.3205/iprs000105

**Published:** 2017-02-16

**Authors:** Reinhard Dolp, Doris Henne-Bruns

**Affiliations:** 1 Department of General and Visceral Surgery, University Hospital Ulm, Germany

**Keywords:** colon penetration, colon perforation

## Abstract

We report of a 57-year-old female patient who underwent a hemipelvectomy with a hemipelvic replacement in 1992. In 2013 the implanted material had to be partially removed due to a periprothetic infection. At that time a palacos spacer was implanted which penetrated two years later into the colon cavity.

## Zusammenfassung

Wir berichten über eine 57-jährige Patientin, bei der 1992 eine Hemipelvektomie mit Beckenteilersatz durchgeführt wurde. 2013 musste das Implantat partiell auf Grund einer periprothetischen Infektion entfernt werden. Es wurde als Platzhalter ein Palacos-Spacer implantiert, der zwei Jahre später in das Kolon penetrierte.

## Introduction

Penetration of implanted material has been reported after different surgical procedures like endovascular aortic repair (aortoenteric fistula), gastric banding (penetration into the stomach) or total hip replacements (penetration into the pelvis). Here, we report a case of colon penetration after a total hip replacement.

## Case description

A 57-year-old female patient presented in 2/2015 in our outpatient clinic with mild, but therapy-refractory constipation since four weeks.

This patient received a right inner hemipelvectomy with a hemipelvic replacement in 1992 because of a malignant schwannoma of the right iliac bone. In 1995, the patient suffered a traumatic right-sided femur fracture, which was treated with a plate osteosynthesis. During the following years she developed a non-irritated chronic fistula underneath her surgical scar. 

In 2013, the chronic fistula became infected and cumulated in a deep gluteal abscess (measuring 4x4 cm) extending to the hemipelvic prosthesis. An explantation of the pelvic replacement was recommended but was initially declined by the patient. She received antibiotics in concordance with the wound culture and sensitivity swab.

**Figure 1:**
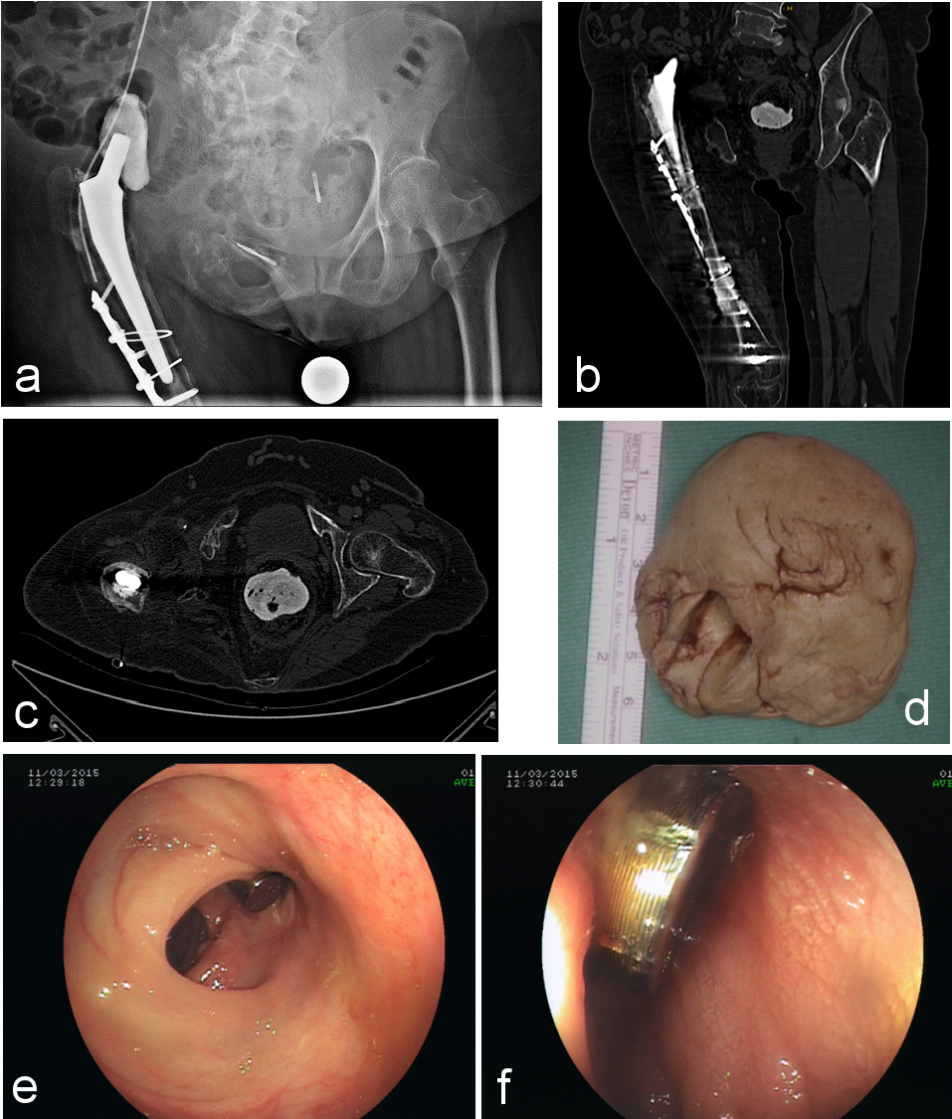
a: Acetabular cab in place; b and c: acetabular cap in the rectum; d: acetabular cap encapsulated by fibrous tissue; e and f: colonoscopy showing the tip of the endoprothesis

In 2014, the explantation of the hemipelvic replacement had to be performed along with the removal of the femoral plate osteosynthesis due to a periprosthetic fracture. She received a 12-hole locking compression plate (LCP), three wire cerclages, a Palacos^®^ spacer, as well as a gentamycin chain (Figure 1a (Fig. 1)). Temporary vacuum dressings were substituted by a silicone capillary drain (easyflow) together with secondary wound closure, leading to a stable situation without pain and mobilisation with a wheeled walker. The two remaining drainages collected a constant putrid secretion, but the patient refused any further surgical therapy.

In March 2015 a follow-up computed tomography (CT) showed the head of the implanted Palacos^®^ spacer inside her rectal cavity (Figure 1b and c (Fig. 1)). With a four-week history of mild constipation as the only symptom she presented with a soft and painless abdomen.

The first step in our department was the removal of the foreign body under general anesthesia through the anus without complications (Figure 1d (Fig. 1)). Afterwards a colonoscopy was performed showing the tip of the endoprothesis 3 cm aboral of the ileocecal region (Figure 1e and f (Fig. 1)).

**Figure 2:**
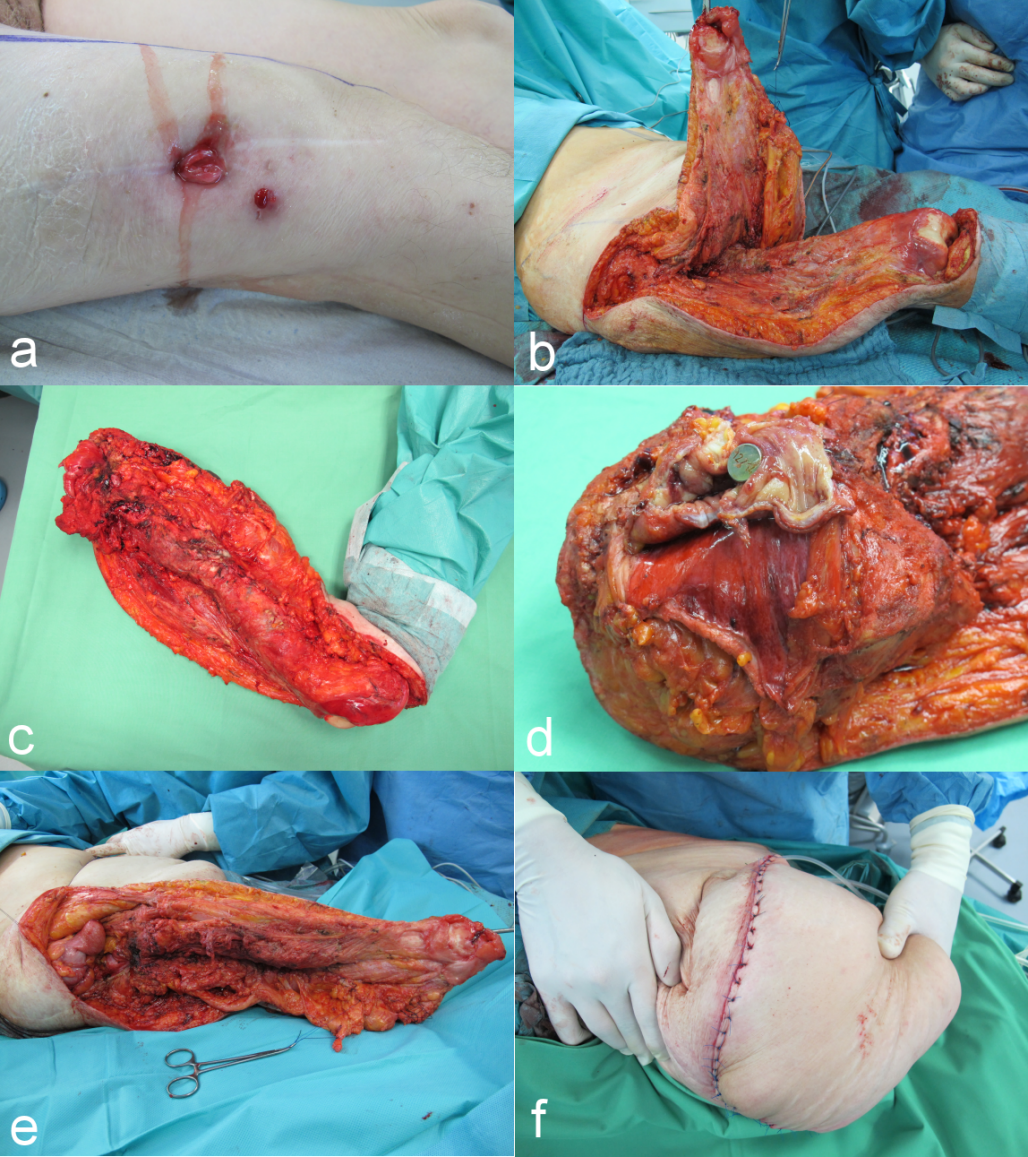
a: Fistulas to the right distal thigh; b: preparation of the myo-cutaneous flap; c: specimen (right leg with adjacent wedge resection of the colon); d: tip of the endoprothesis within the colon; e: final myo-cutaneous flap; f: result at the end of the operation

Due to the chronic infection of the prosthesis with several fistulas to the gluteal region as well as to the lateral thigh (Figure 2a (Fig. 2)) and the penetration into the right colon we decided to perform a en bloc resection combining a right-leg amputation with a wedge resection of the right colon. Wound coverage was achieved by a large muscular cutaneous flap from the vastus, the rectus femoris and the adductal muscles (Figure 2b–f (Fig. 2)).

The postoperative course was uneventful. After three weeks, and with intensive physiotherapy, she was completely mobile with the use of a wheelchair and could be discharged.

A bowel perforation by implanted foreign material (e.g. after gastric banding, implantation of orthopedic prothesis) is a rare complication. The complete absence of symptoms or only mild symptoms accompanying this process is even rarer. The penetration of foreign material into the gut usually causes severe complications like pain, melena respectively hematemesis or sepsis like in case of aortoenteric fistulas after endovascular repair via prosthesis [1]. There are few case reports with asymptomatic bowel perforations for example due to a peritoneal dialysis catheter (Tenckhoff^®^) [2]. However, in the here presented case the head of the spacer that penetrated into the colon is relatively large (6x5 cm) in comparison with the foreign bodies described in the literature before.

## Notes

### Competing interests

The authors declare that they have no competing interests.
